# Effects of heat stress on growth performance, carcass traits, serum metabolism, and intestinal microflora of meat rabbits

**DOI:** 10.3389/fmicb.2022.998095

**Published:** 2022-11-28

**Authors:** Hongli Liu, Bin Zhang, Fan Li, Lei Liu, Tongao Yang, Haihua Zhang, Fuchang Li

**Affiliations:** ^1^Shandong Provincial Key Laboratory of Animal Biotechnology and Disease Control and Prevention, Department of Animal Science and Technology, Shandong Agricultural University, Taian, Shandong, China; ^2^Hebei Key Laboratory of Specialty Animal Germplasm Resources Exploration and Innovation, Department of Animal Science and Technology, Hebei Normal University of Science and Technology, Qinhuangdao, Hebei, China

**Keywords:** rabbits, heat stress, growth performance, metabolomics, microbiome

## Abstract

To investigate the effects of heat stress on meat rabbits, we assigned 80 rabbits to the moderate temperature group (24 ± 1°C; Control group) and the continuous high-temperature group (HT group), then monitored the effects using growth performance, carcass characteristics, biochemical assays, UPLC–MS/MS-based metabolomics, and microbiome. The results showed that after continuous high-temperature exposure, the average daily gain, average daily feed intake, and thymus index were significantly decreased (*p* < 0.05). Contents of HSP70, ALP, and Cortisol in serum were significantly increased, while TP, GLU, T3, and T4 were significantly decreased (*p* < 0.05). Nine kinds of differential metabolites were screened by serum metabolomics, which can be used as biomarkers of heat stress in meat rabbits. The selected differential metabolites were analyzed by KEGG annotation and enrichment analysis. The results showed that 14 pathways affected by heat stress were identified by KEGG pathway enrichment analysis, including Sphingolipid metabolism, Pyrimidine metabolism, Citrate cycle (TCA cycle)), aminoacyl-tRNA biosynthesis, and so on. The analysis of the effect of heat stress on the cecal microflora of meat rabbits showed that the abundance of cecal Proteus in the HT group was significantly higher than that in the moderate Control group. The number of Candidatus-saccharimonas in the cecum microflora was significantly higher than that in the moderate temperature group (*p* < 0.05) which may be related to inflammatory diseases in the heat stress group. These findings indicated that the heat-stressed rabbits were in negative energy balance, which affected protein metabolism, and subsequently affected growth performance and carcass characteristics.

## Introduction

With the continuous acceleration of global warming and intensive breeding environment, heat stress has gradually become a global issue of common concern in modern livestock and poultry production, and heat stress is considered to be one of the most important stresses in livestock and poultry breeding. Heat stress refers to the sum of non-specific responses produced by animals when they are stimulated by excessive temperature beyond their thermoregulatory capacity (Mount, 1975). Heat stress causes serious economic losses to the livestock industry by changing various physiological and biochemical reactions of the body, reducing feed intake, daily gain, feed conversion rate, and product quality (West, 2003; Franco, 2004; Beckford et al., 2020).

Climate warming is unequivocal, and will greatly impact animal health and growth, especially during the summer months, either directly or secondarily (Leon et al., 2005). Heat stress seriously restricts the development of the rabbit industry. Rabbits belong to constant temperature animals, and the normal body temperature is 38.5–39.9°C, while the upper limit of the moderate temperature of rabbits is lower than 30°C in all current studies. The suitable growth temperature of meat rabbits is 15–25°C. The heat insulation layer formed by a few sweat glands and thick hair on the body surface of rabbits leads to poor heat dissipation ability (Lee, 1965). Moreover, the rabbit is very sensitive to temperature change and is easy to produce stress reactions. When the ambient temperature is higher than the body surface temperature of rabbits, non-evaporative heat dissipation methods such as radiation, convection and conduction fail, and respiratory heat dissipation becomes the main way of heat dissipation in rabbits. However, after all, the ability of breathing and heat dissipation is limited, and high temperature for a long time will increase the respiratory frequency of rabbits, wheeze, increase body temperature, and then appear heat stress reaction, resulting in changes in rabbit behavior and physiology. Heat stress can cause changes in some of the physiological indicators of meat rabbits such as rectal temperature increase, feed intake and daily gain decreased, and water consumption increased, thus heat stress will increase energy consumption, reduce production performance, and reproductive performance. The thermoregulation characteristics of rabbits make them have poor tolerance to a high heat environment, which is related to breed, age, sex, stress intensity, and duration (Yang et al., 2011; Li et al., 2019; Zheng et al., 2022). Heat stress resulted in a decrease in feed intake, daily gain, and meat quality in rabbits (Marai et al., 2002). Research has found that, heat stress regulates stress response through the endocrine system (Mete et al., 2012).

The present study aimed to characterize the serum metabolic profile of heat-stressed broilers and to investigate the change rule and related regulatory mechanisms using Ultra Performance Liquid Chromatography Tandem Mass Spectrometry (UPLC–MS/MS) metabolomics, biochemical validation, microbiomics, and multivariate data analysis. The study also aimed to elucidate the relationships between growth performance, serum biochemical and hormonal parameters, serum metabolism, and gut microflora changes in heat-stressed rabbits.

## Materials and method

### Animal and experiment

**Figure 1:**
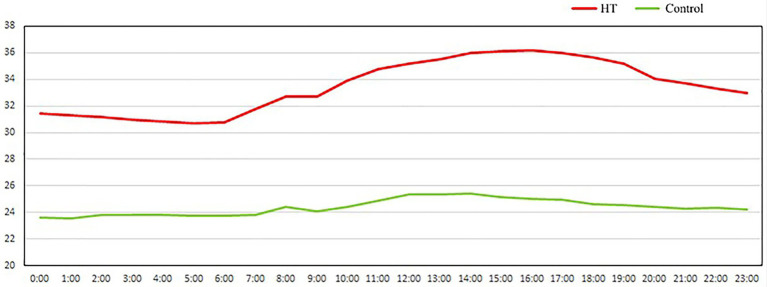
The actual average temperature in the room every day.

In the experiment, eighty 35-day-old Hyla meat rabbits (half male and half female) were pre-fed at 24 ± 1°C for 7 days, and then sixty 42-day-old meat rabbits were randomly divided into two groups: moderate temperature group (24 ± 1°C) and continuous high-temperature group (high-temperature feeding, 34 ± 2°C). The temperature and humidity were adjusted by the air conditioner and humidifier (ZDR-41). Hangzhou Zeda Instrument Co., Ltd.) automatically records room temperature every 30 min, the experimental period is 14 days (Figure 1). Rabbits were housed in homemade plastic cages (60 × 40 × 40 cm) in couples. The relative humidity and photoperiod were maintained according to commercial conditions (55°C to 65°C, 12 light/12 dark). Diets were pelleted using pressure, and the diameter of the pellets was 4 mm. All rabbits had free access to feed and water during the rearing period. At the end of the experimental period, 8 rabbits in each group were randomly selected to fast for 12 h, and the blood was collected from the heart. The blood was placed for 30 min and centrifuged at 3,000 rpm for 10 min. The serum was separated and stored at-80°C for blood biochemical index and metabonomics determination. After blood collection, the rabbits were killed after breaking their necks and weighed after peeling off the fur, thymus, spleen, liver, kidney, shoulder fat, and perirenal fat. Samples of cecal contents were quickly frozen in liquid nitrogen and stored at-80°C for 16 s analysis of microbial diversity and metabonomics analysis.

### Growth performance and sample collection

Rabbits in every replicate from each treatment group were weighed both on the transfer days (experimental 8-day) and at the end of the experiment (experimental 21-day). Average daily gain (ADG), Average daily feed intake (ADFI), Feed conversion ratio (FCR), Liver index, Spleen index, Thymus index, Kidney index, Shoulder fat rate, and Kidney fat rate were calculated according to the formula:

ADG = body increase (g)/number of trial days.

ADFI = (feed intake-leftover feed) /number of trial days.

FCR = ADFI/ADG.

Fully eviscerated slaughtering rate (%) = full eviscerated weight/live weight before slaughter * 100.

Liver index (%) = liver weight/live weight before slaughter * 100.

Spleen index (%) = spleen weight / live weight before slaughter * 100

Thymus index (%) = thymus weight / live weight before slaughter * 100

Kidney index (%) = kidney weight / live weight before slaughter * 100

Shoulder fat rate (%) = shoulder fat weight/live weight before slaughter * 100.

Kidney fat rate (%) = perirenal fat weight/live weight before slaughter * 100.

### Serum biochemical and hormonal parameters

Serum concentrations of total protein (TP), albumin (ALB), glucose (GLU), triglyceride (TG), cholesterol (CHO), urea (UREA), high-density lipoprotein (HDL), low-density lipoprotein (LDL), calcium (Ca), phosphorus (P), glutamic pyruvic transaminase (ALT), and glutamic-oxalacetic transaminase (AST) were measured using a Hitachi 7,020 automatic biochemical analyzer (Hitachi Ltd., Tokyo, Japan). Serum growth hormone (GH), thyroid-stimulating hormone (TSH), triiodothyronine (T3), and tetraiodothyronine (T4) were determined by the ELISA method with the corresponding ELISA kits (Ybio Bio-tech Co., Ltd., Shanghai, China).

### Metabonomic analysis based on UPLC–MS/MS

The serum samples were taken from the refrigerator at-80°C and thawed in the refrigerator at 4°C. Six samples were selected for each group. 100 and 400 μl pre-cooled 80% methanol containing 0.1% formic acid were mixed by rotation method, and the samples were incubated for 5 min on ice. Under the condition of 15,000 g 10 min at 4°C, the supernatant was injected into the sample by centrifugation and analyzed by LC–MS/MS system. The materials used for LC-MS/MS analyses were given in Supplementary material S1 (Liu et al., 2022b).

### Gut microbiomics

Microbial community genomic DNA was extracted from cecal contents samples using the E.Z.N.A.® soil DNA Kit (Omega Bio-tek, Norcross, GA, U.S.) according to the manufacturer’s instructions. The materials used for DNA extraction, PCR amplification of 16S rRNA gene, and 16S rRNA gene sequencing reads were given in Supplementary material S2 (Liu et al., 2022a).

The DNA extract was checked on 1% agarose gel, and DNA concentration and purity were determined with NanoDrop 2000 UV–vis spectrophotometer (Thermo Scientific, Wilmington, United States). The hypervariable region V3-V4 of the bacterial 16S rRNA gene was amplified with primer pairs 338F (5′-ACTCCTACGGGAGGCAGCAG-3′) and 806R (5′-GGACTACHVGGGTWTCTAAT-3′) by an ABI GeneAmp® 9,700 PCR thermocycler (ABI, CA, United States).

The PCR amplification of 16S rRNA gene was performed as follows: initial denaturation at 95°C for 3 min, followed by 27 cycles of denaturing at 95°C for 30 s, annealing at 55°C for 30 s and extension at 72°C for 45 s, and single extension at 72°C for 10 min, and end at 4°C. The PCR mixtures contain 5 × TransStart FastPfu buffer 4 μl, 2.5 mM dNTPs 2 μl, forward primer (5 μM) 0.8 μl, reverse primer (5 μM) 0.8 μl, TransStart FastPfu DNA Polymerase 0.4 μl, template DNA 10 ng, and finally ddH2O up to 20 μl. PCR reactions were performed in triplicate. The PCR product was extracted from 2% agarose gel and purified using the AxyPrep DNA Gel Extraction Kit (Axygen Biosciences, Union City, CA, United States) according to the manufacturer’s instructions and quantified using Quantus™ Fluorometer (Promega, United States).

Purified amplicons were pooled in equimolar and paired-end sequenced on an Illumina NovaSeq PE250 platform (Illumina, San Diego, United States) according to the standard protocols by Majorbio Bio-Pharm Technology Co. Ltd. (Shanghai, China). The raw reads were deposited into the NCBI Sequence Read Archive (SRA) database.

The raw 16S rRNA gene sequencing reads were demultiplexed, quality-filtered by fastp version 0.20.0 and merged by FLASH version 1.2.7 with the following criteria: (i) the 300 bp reads were truncated at any site receiving an average quality score of <20 over a 50 bp sliding window, and the truncated reads shorter than 50 bp were discarded, reads containing ambiguous characters were also discarded; (ii) only overlapping sequences longer than 10 bp were assembled according to their overlapped sequence. The maximum mismatch ratio of overlap region is 0.2. Reads that could not be assembled were discarded; (iii) Samples were distinguished according to the barcode and primers, and the sequence direction was adjusted, exact barcode matching, 2 nucleotide mismatch in primer matching.

Operational taxonomic units (OTUs) with 97% similarity cutoff were clustered using UPARSE version 7.1, and chimeric sequences were identified and removed. The taxonomy of each OTU representative sequence was analyzed by RDP Classifier version 2.2 against the 16S rRNA database (eg. Silva v138) using the confidence threshold of 0.7.

## Data processing and statistical analysis

### Production performance and serum biochemical data

The raw data were statistically processed using Excel and the data were analyzed using the One-Way ANOVA model in SAS 8.2 software and compared using Duncan’s multiple tests. *p*-values were obtained, with *p* < 0.05 indicating significant differences, and results are presented as means ± SEs.

### Metabolomics data processing and analysis

Statistical analyses were performed using the statistical software R (R version R-3.4.3), Python (Python version 2.7.6) and CentOS (CentOS version 6.6), and when the data were not normally distributed, they were transformed normally using the area normalization method. The metabolites were annotated using the KEGG database^1^ and the HMDB database^2^ and Lipidmap database.^3^ Principal component analysis (PCA) and partial least squares discriminant analysis (PLS-DA) were performed on MetaX. Statistical significance was calculated using a univariate analysis of variance (T-test) *p* < 0.05.

### Microbial diversity data processing and analysis

Quality control of raw sequenced sequences using fastp (https://github.com/OpenGene/fastp, version 0.20.0) software and FLASH (http://www.cbcb.umd.edu/software/flash, version 1.2.7) software for splicing. OTU clustering of sequences based on 97% similarity using UPARSE software (http://drive5.com/uparse/, version 7.1). Each sequence was annotated for species classification using the RDP classifier (http://rdp.cme.msu.edu/, version 2.2), compared to the Silva 16S rRNA database (v132), and a comparison threshold of 70% was set.

## Results

### Effect of heat stress on production performance of meat rabbits

Table 1 showed that under the condition of no difference in initial body weight, the final weight, ADFI and ADG of meat rabbits in the heat stress group were significantly lower than those in the control group (*p* < 0.05). At the same time, the thymus index and liver index in the heat stress group were significantly lower than those in the control group (*p* < 0.05), while the shoulder fat rate and kidney fat rate in the heat stress group were significantly higher than those in the control group (*p* < 0.05).

**Table 1:** Effect of heat stress on meat rabbit’s carcass trait.

Items	Control	HT	*p*-value
Initial weigh (kg)	1.19 ± 0.02	1.21 ± 0.03	0.5679
Final weight of test (kg)	1.62 ± 0.02^a^	1.54 ± 0.03^b^	0.0240
ADFI (g)	133.28 ± 2.76^a^	107.21 ± 2.30^b^	<0.001
ADG (g)	31.65 ± 1.32^a^	26.10 ± 1.26^b^	0.0036
FCR	4.43 ± 0.17	4.30 ± 0.22	0.6322
Eviscerated rate (%)	46.03 ± 0.51	47.18 ± 0.84	0.2521
Thymus index (%)	0.21 ± 0.01^a^	0.17 ± 0.01^b^	0.0282
Spleen index (%)	0.08 ± 0.01	0.07 ± 0.01	0.0834
Liver index (%)	2.94 ± 0.08^a^	2.66 ± 0.09^b^	0.0326
Kidney index (%)	0.67 ± 0.02	0.64 ± 0.02	0.2505
Shoulder fat percentage (%)	0.15 ± 0.01^b^	0.20 ± 0.02^a^	0.0482
Kidney fat rate (%)	0.25 ± 0.01^b^	0.37 ± 0.05^a^	0.0270

Values are means ± SEMs; *n* = 8. ^ab^Values with different superscripts differ significantly (*p* < 0 0.05).

### Effects of heat stress on serum biochemical indexes of meat rabbits

The effect of heat stress on serum biochemical indexes of meat rabbits is shown in Table 2. Compared with the control group, the contents of HSP70, ALP and cortisol in the serum of the heat stress group increased significantly (*p* < 0.05), while the contents of TP, GLU, T3 and T4 decreased significantly (*p* < 0.05), and other biochemical indexes did not change significantly.

**Table 2:** Effects of Heat stress on serum biochemical of meat rabbits.

Items	Control	HT	*p*-value
HSP70 (pg/ml)	1403.61 ± 32.10^b^	1569.05 ± 14.48^a^	0.0015
Cortisol (ng/L)	19.48 ± 1.60^b^	52.39 ± 5.81^a^	0.0003
TP (g/L)	64.50 ± 1.83^a^	58.63 ± 1.05^b^	0.0194
ALB (g/L)	30.60 ± 0.47	29.37 ± 1.03	0.3029
UREA (mmol/L)	7.37 ± 0.44	8.25 ± 0.18	0.0967
GLU (mmol/L)	7.45 ± 0.21^a^	6.84 ± 0.1^b^	0.0269
TG (mmol/L)	1.38 ± 0.32	0.72 ± 0.10	0.0816
Ca (mmol/L)	4.26 ± 0.08	4.53 ± 0.12	0.0918
P (mmol/L)	3.28 ± 0.12	2.85 ± 0.20	0.0938
HDL (mmol/L)	0.43 ± 0.04	0.61 ± 0.09	0.0875
LDL (mmol/L)	0.46 ± 0.08	0.50 ± 0.02	0.5943
T3 (ng/ml)	0.41 ± 0.06^a^	0.13 ± 0.03^b^	0.0014
T4 (μg/dl)	1.39 ± 0.21^a^	0.29 ± 0.12^b^	0.0011

Values are means ± SEMs; *n* = 8. ^ab^Values with different superscripts differ significantly (*p* < 0.05).

### Effects of heat stress on serum metabolomics in meat rabbits

Metabolome refers to a collection of small molecule compounds that are involved in the metabolism of organisms and maintain normal growth and development of organisms. These are mainly referred to as endogenous small molecules with relative molecular weight of less than 1,000. We used LC–MS/MS method combined with positive ion mode (POS) and negative ion mode (NEG) for serum metabolite analysis, making metabolite coverage higher and the detection effect better. In POS mode, a total of 5,456 peaks were obtained, and 343 substances of these were identified while the number of metabolites annotated to the public database was 399. In NEG mode, a total of 4,809 peaks were obtained, and 496 substances of these were identified and 464 were annotated to the public database (see Table 3).

**Table 3:** TOTAL ion count and identification statistics table.

Ion mode	All peaks	Identified metabolites	Metabolites in Library
POS	5,456	343	299
NEG	4,809	496	464

**Figure 2:**
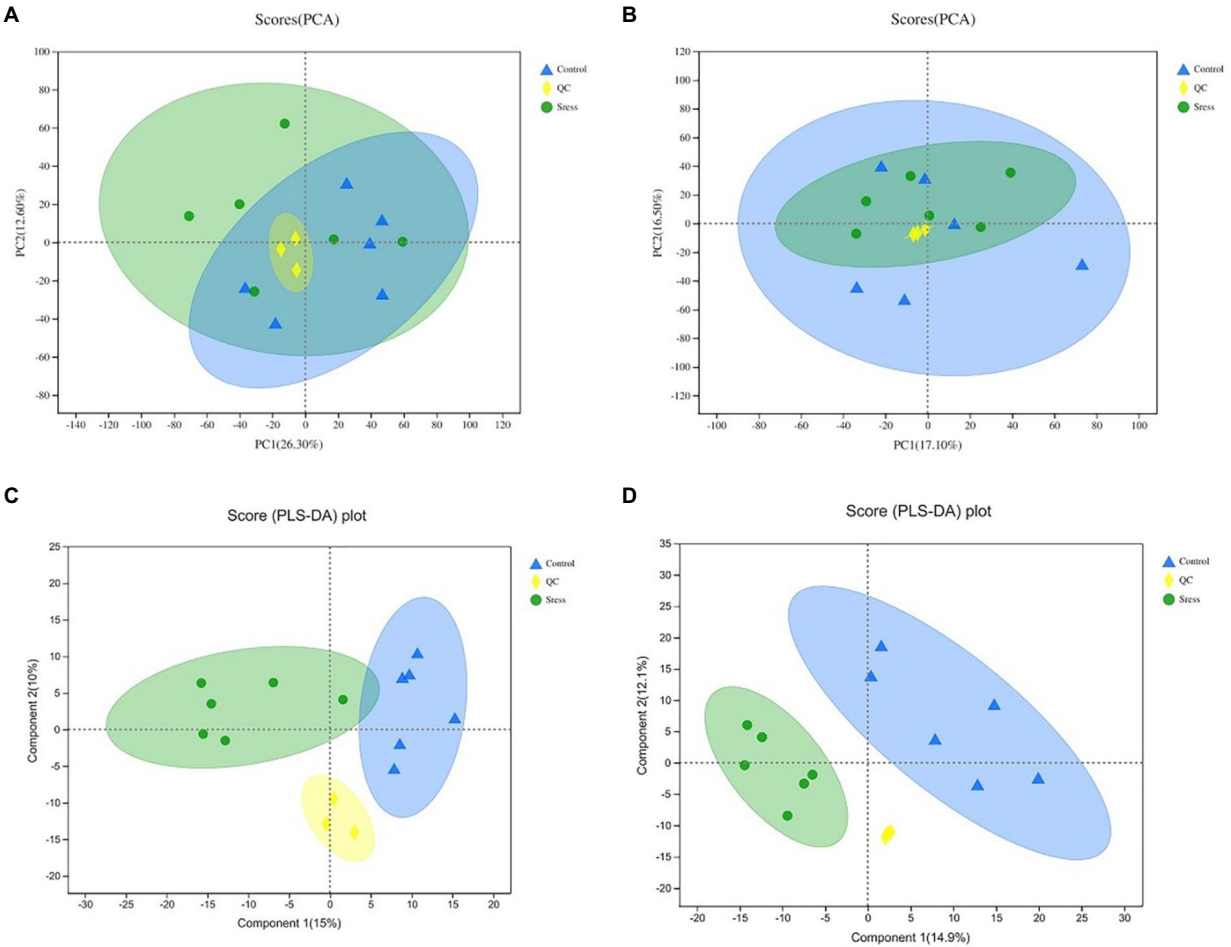
PCA and PLS-D Aanalysis score of quality control under the positive ion mode **(A,C)** and negative ion mode **(B,D)**. In the figure, Control represents the appropriate temperature group, and Stress represents the heat stress group, *n*=6.

According to the expression of metabolites in different samples, PCA principal component analysis was carried out to evaluate the similarity of samples within groups and the differences between samples, and PLS-DA analysis was carried out. The results are shown in Figure 2. The PCA and PLS-DA score maps show that the QC samples are gathered together, and PCA shows that the confidence ellipses of the two groups of samples overlap, but there are no outliers, indicating that the degree of variability between the two groups of samples is small, and the results of PLS-DA analysis show that the two groups of samples are separated greatly, indicating that the classification effect is significant.

**Figure 3:**
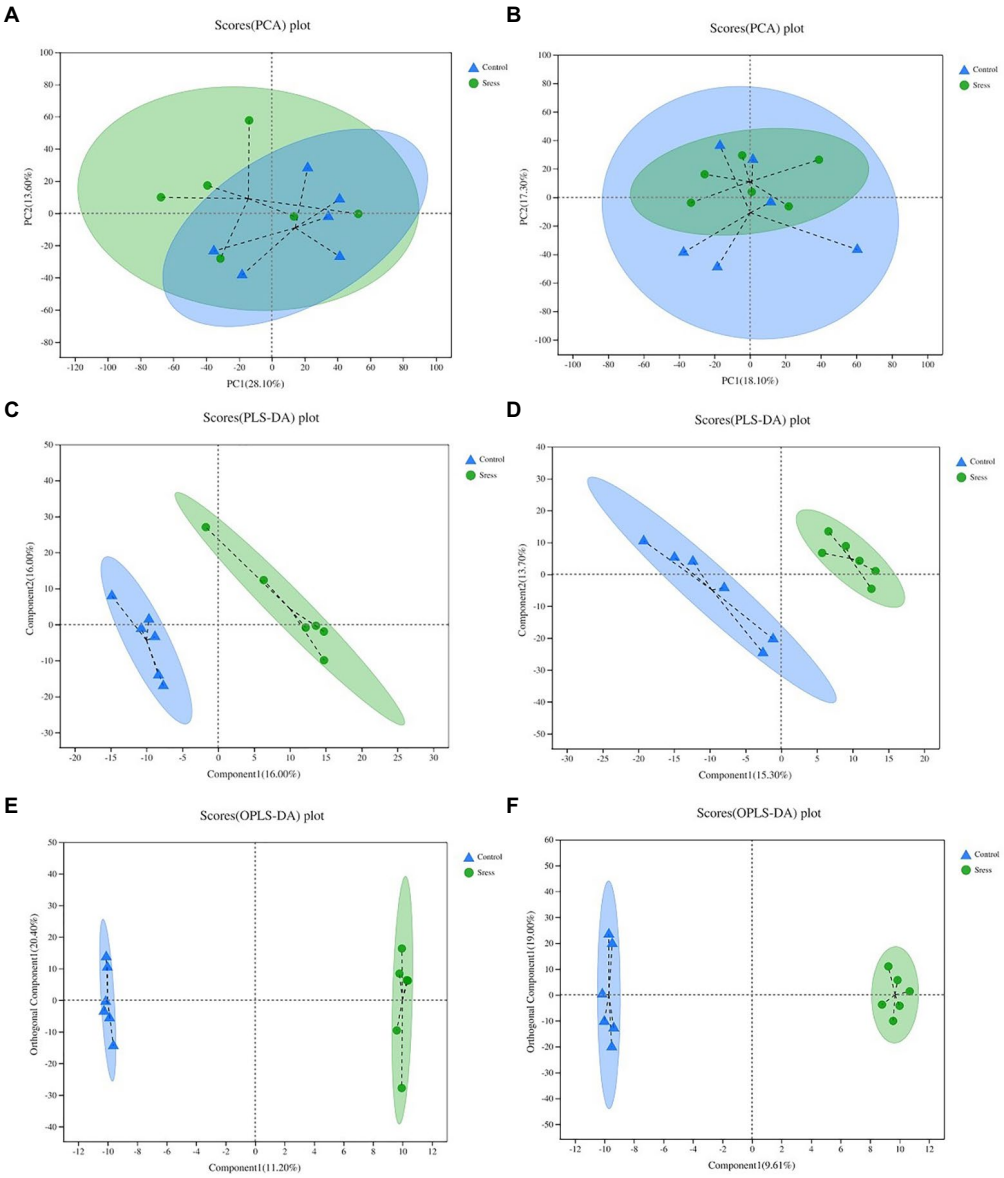
PCA, PLS-DA, OPL-DA analysis under the positive ion mode **(A,C,E)** and negative ion mode **(B,D,F)**. In the figure, Control represents the appropriate temperature group, and Stress represents the HT group, *n*=6.

Multidimensional statistical analysis of the identified metabolites was carried out by PCA, PLS-DA and OPLS-DA statistical analysis (Figure 3) to screen out the most important and noteworthy differential metabolites. In the PCA model, the sample distances between the two sample groups overlap, indicating that the intra-and inter-group differences between the two groups are small. The PLS-DA score chart shows that the separation degree of the two groups of samples is high, indicating that the classification effect is significant. The OPLS-DA score map filters out the information that has nothing to do with the grouping through orthogonal rotation so that it can better distinguish the differences between groups and improve the efficiency of the model. It can be seen from the chart that the classification effect between the two groups of samples is significant, which shows that the construction of the experimental model is reliable and the data is effective.

**Figure 4:**
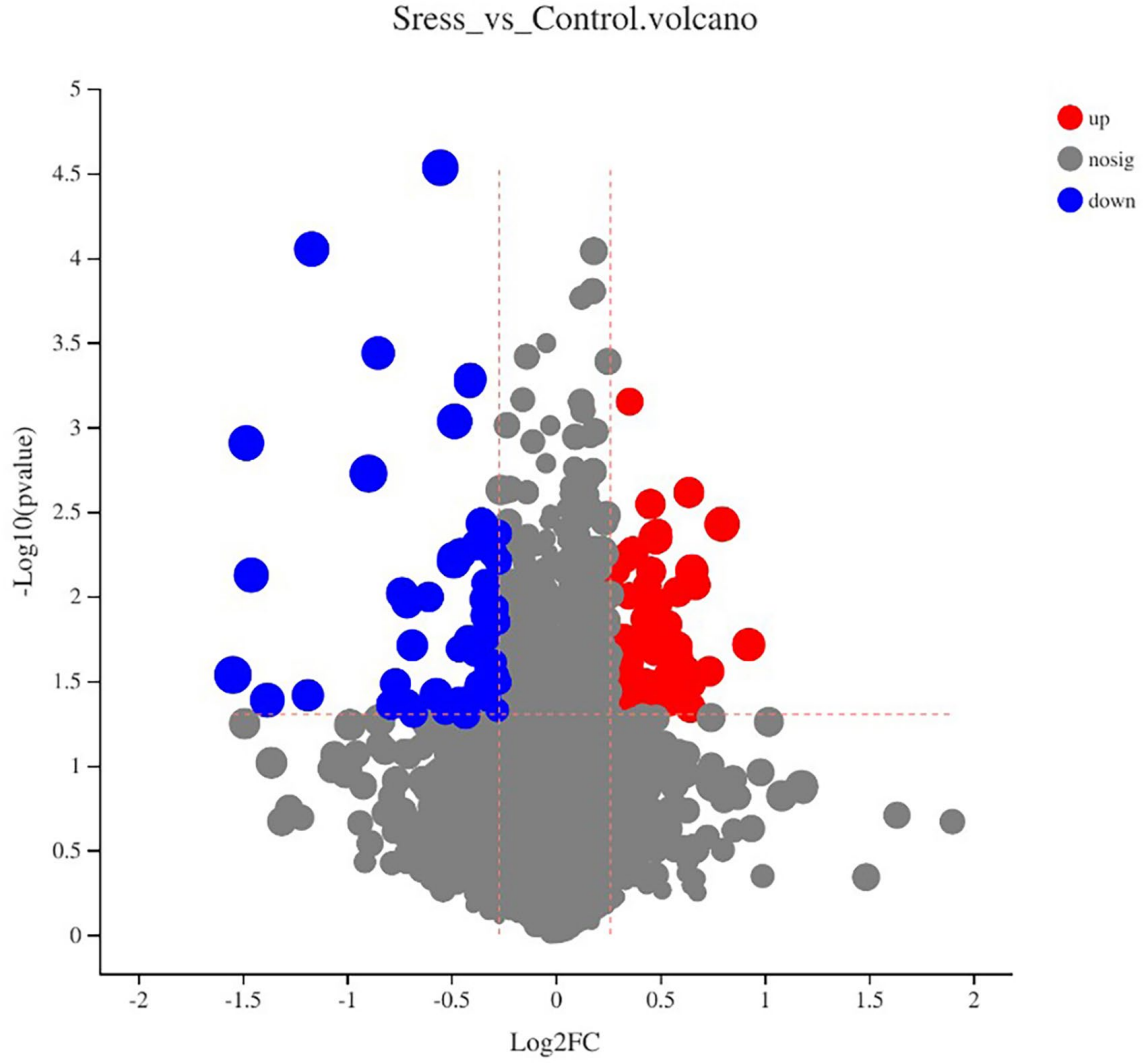
Volcano plot by metabolomics analysis.

Multidimensional statistical analysis showed that VIP > 1 and FC > 1.2 or FC < 0.83 were used as screening criteria for differential metabolites. A total of 126 differential metabolites were screened, including 48 up-regulated differential metabolites and 78 down-regulated differential metabolites, while there are 9 different metabolites with specific names (volcanic diagram see Figure 4, results see Table 4). For all the identified metabolites, FC and T-tests were combined to measure the influence and explanatory ability of the expression patterns of metabolites on the classification and discrimination of each group of samples according to the VIP values obtained by the PLS-DA model. The differential metabolites with biological significance were mined and selected at the same time.

**Table 4:** Different metabolites in the meat rabbit serum after heat stress.

Metabolite name	VIP	FC	*p*-value	Regulated
Diaminopimelic acid	2.8278	0.5879	0.0330	Down
Sphinganine	2.3686	0.7955	0.0396	Down
Dihydronaringenin-O-sulphate	3.2487	1.8992	0.01937	Up
Erinacine P	2.0796	1.323	0.03228	Up
Cis-4,10,13,16-Docosatetraenoic Acid	3.2878	0.555	0.000367	Down
3-keto-Digoxigenin	2.1074	0.7263	0.02053	Down
Glycinoeclepin B	3.7207	0.3584	0.001247	Down
Ganoderic acid H	3.6173	0.3643	0.00752	Down
2-Methoxyacetaminophen glucuronide	2.1419	0.8201	0.02961	Down

**Figure 5:**
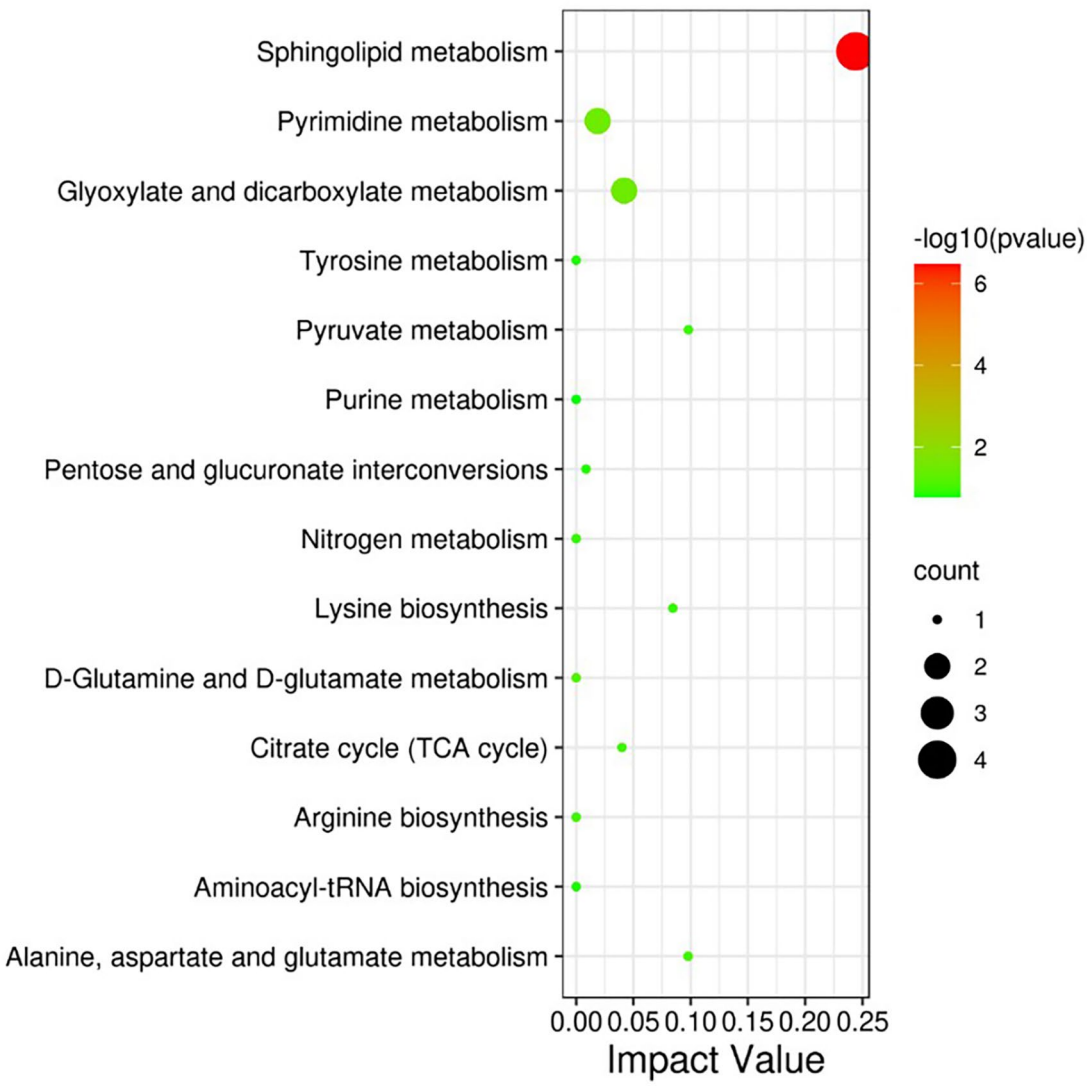
KEGG pathway significantly enriched in differential metabolites. The size of each spot represents the metabolite number, and the color represents -log10 (*p*-value).

The differential metabolites were analyzed by KEGG database and significantly enriched to 14 pathways affected by heat stress (Figure 5), including Phospholipid metabolism pathway, Glyoxylate and dicarboxylate metabolism, Pyrimidine metabolism, Pyruvate metabolism, Alanine, aspartate and glutamate metabolism, Lysine biosynthesis, Citrate cycle (TCA cycle), Aminoacyl-tRNA biosynthesis, etc.

### Effects of heat stress on cecal microflora of meat rabbits

**Figure 6:**
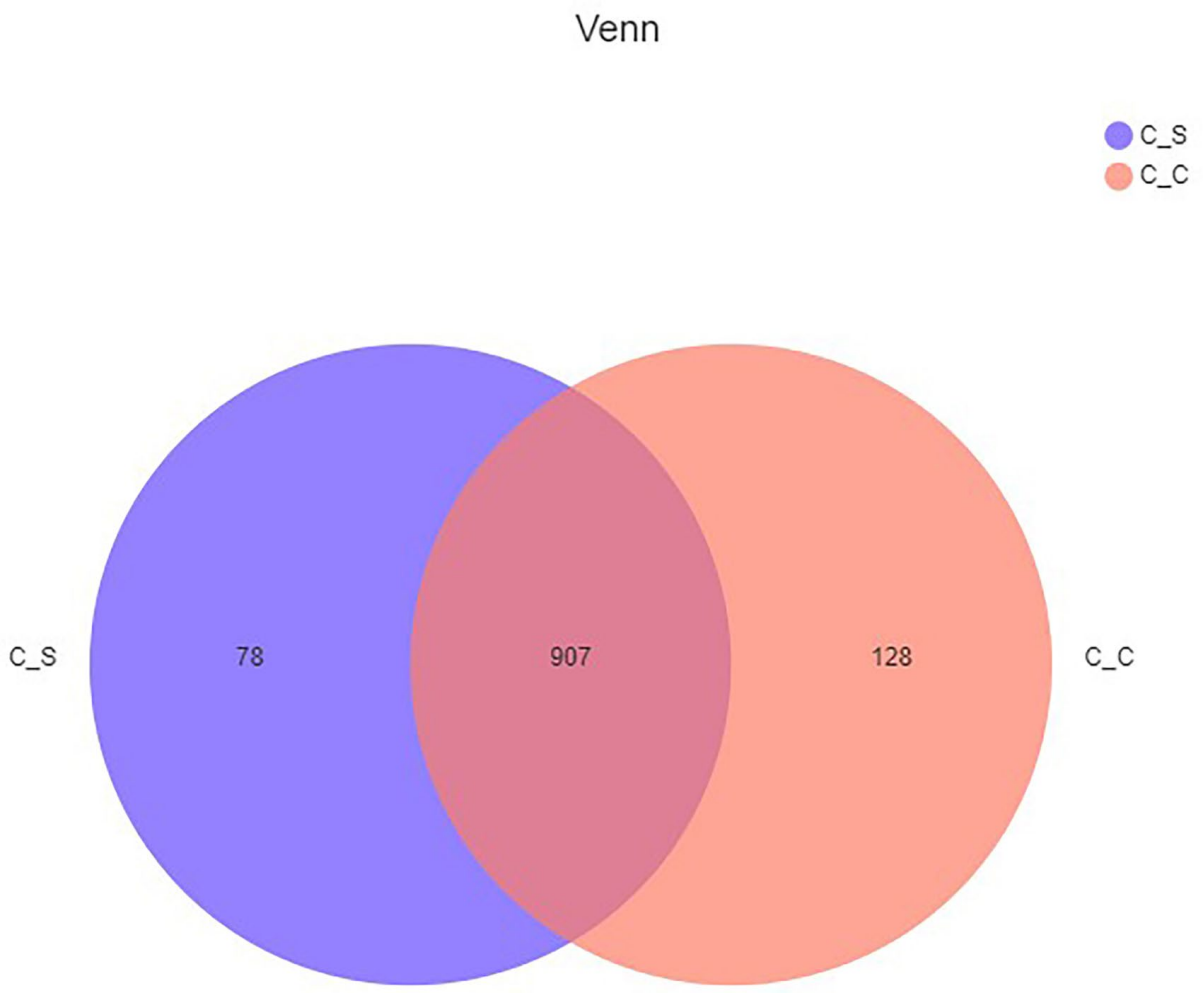
Shared OTU across different groups. In the figure, C▬S represents the HT group and C▬C represents the appropriate temperature group, *n*=6.

Tags were clustered at 97% similarity level to obtain OTU, and taxonomic annotation of OTU based on Silva (bacteria) taxonomic database, and the number of OTU of each sample was obtained. A total of 1,113 different OTU, were obtained from the two groups of samples. The Venn map (Figure 6) showed that there were 907 OTU in the moderate temperature group and heat stress group, 128 in the cecum of meat rabbits in the appropriate temperature group, and 78 OTU in the heat stress group.

**Figure 7:**
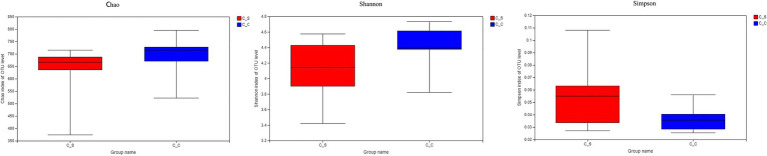
Alpha diversity.

The α diversity of the samples was analyzed by Chao1, Shannon, and Simpson by T-tests. The results show that there is no significant difference between each index in Figure 7, indicating that there is no significant difference in flora diversity, flora abundance and community coverage between the two groups.

**Figure 8:**
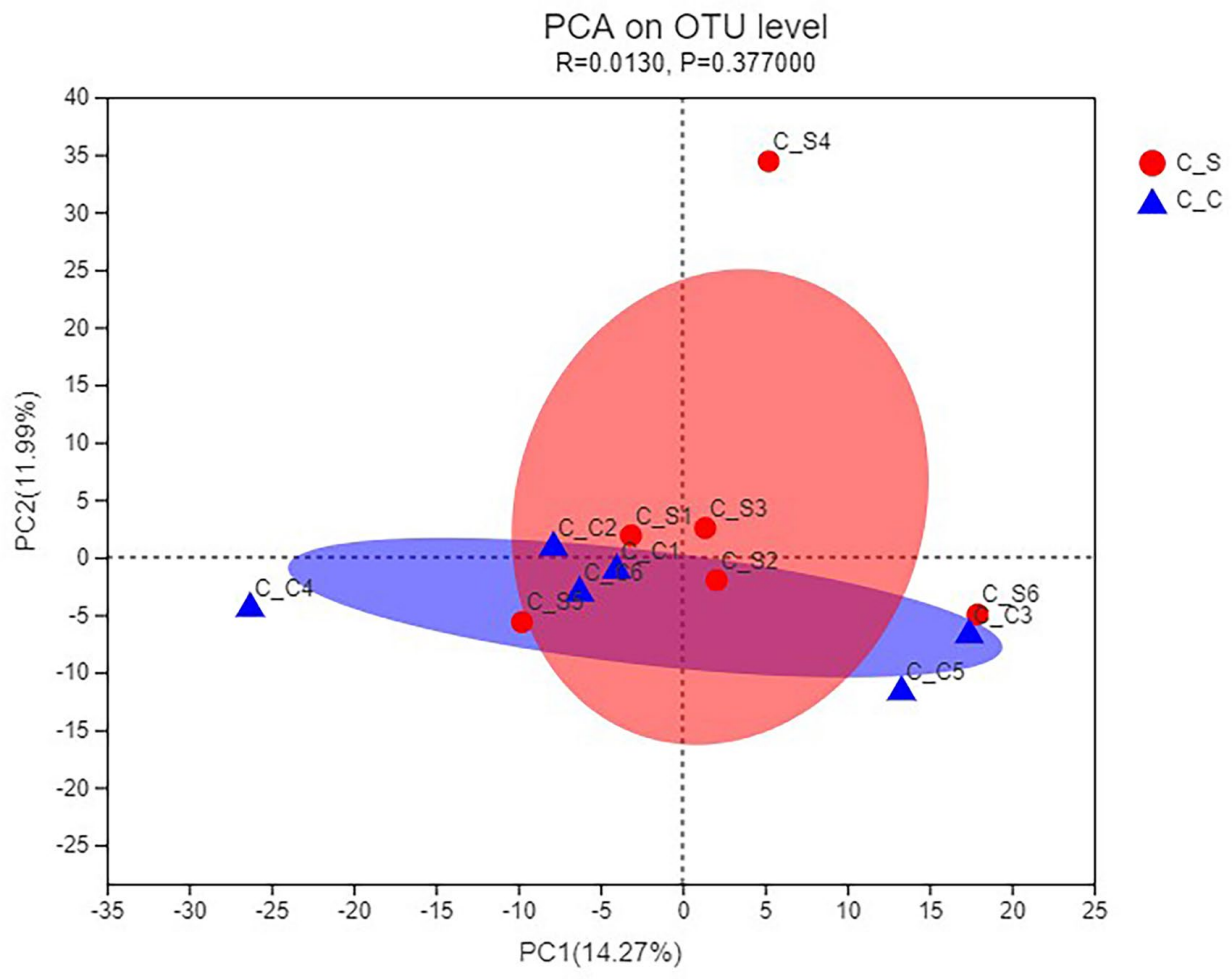
PCA of the cecal microflora of rabbits. In the figure, C▬S represents the HT group and C▬C represents the appropriate temperature group, *n*=6.

As shown in Figure 8, PCA uses the analysis of variance of the OTUs of different samples to present the calculation results on a two-dimensional coordinate graph, and the distance between the samples reflects the differences between the samples. It can be seen that the relative aggregation of the two groups of samples in the picture, PC1 = 14.27%, PC2 = 11.99%, shows that the species composition of the two groups of samples is similar, there is no significant difference.

**Figure 9:**
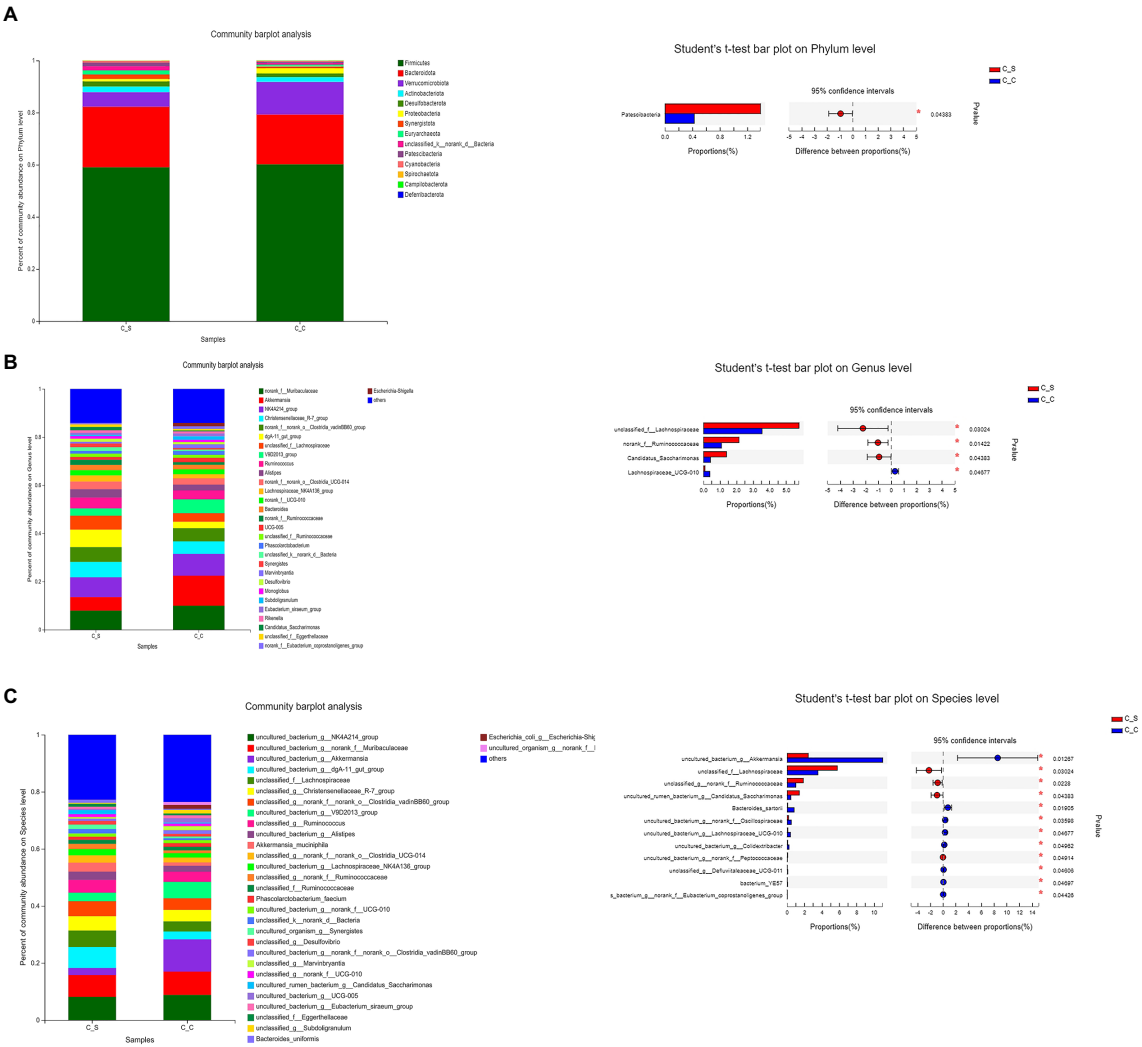
Effects of stress on cecum bacterial in meat rabbits. Relative abundance of species and between-group difference tests at different species taxonomy levels (**A**:Phylum level, **B**:Genus level, **C**:Species level). In the figure, C▬S represents the HT group and C▬C represents the appropriate temperature group, *n*=6.

As shown in Figure 9, the dominant flora in the cecum of meat rabbits was Firmicutes, bacteroidota and verrucomicobiota, while the Proteobacteri of Proteus in cecum of meat rabbits under heat stress was significantly higher than that in the temperature group (*p* < 0.05). Further refinement of the taxonomy to genus level, Muribaculaceae unnamed genera, Ackermania are the dominant genera. At the species level, the abundance of akkermansia_muciniphila increased. The results of the T-test in the cecum of heat-stressed meat rabbits showed that the number of Lachnospiraceae-UGG-010 in the intestines of heat-stressed meat rabbits was significantly lower than that in the normal temperature group, while the unnamed genera of Lachnospiraceae, Ruminococcaceae and Candidatus-saccharimonas in the heat-stressed meat rabbits were significantly higher than those in the cecum samples of the heat-stressed meat rabbits.

**Figure 10:**
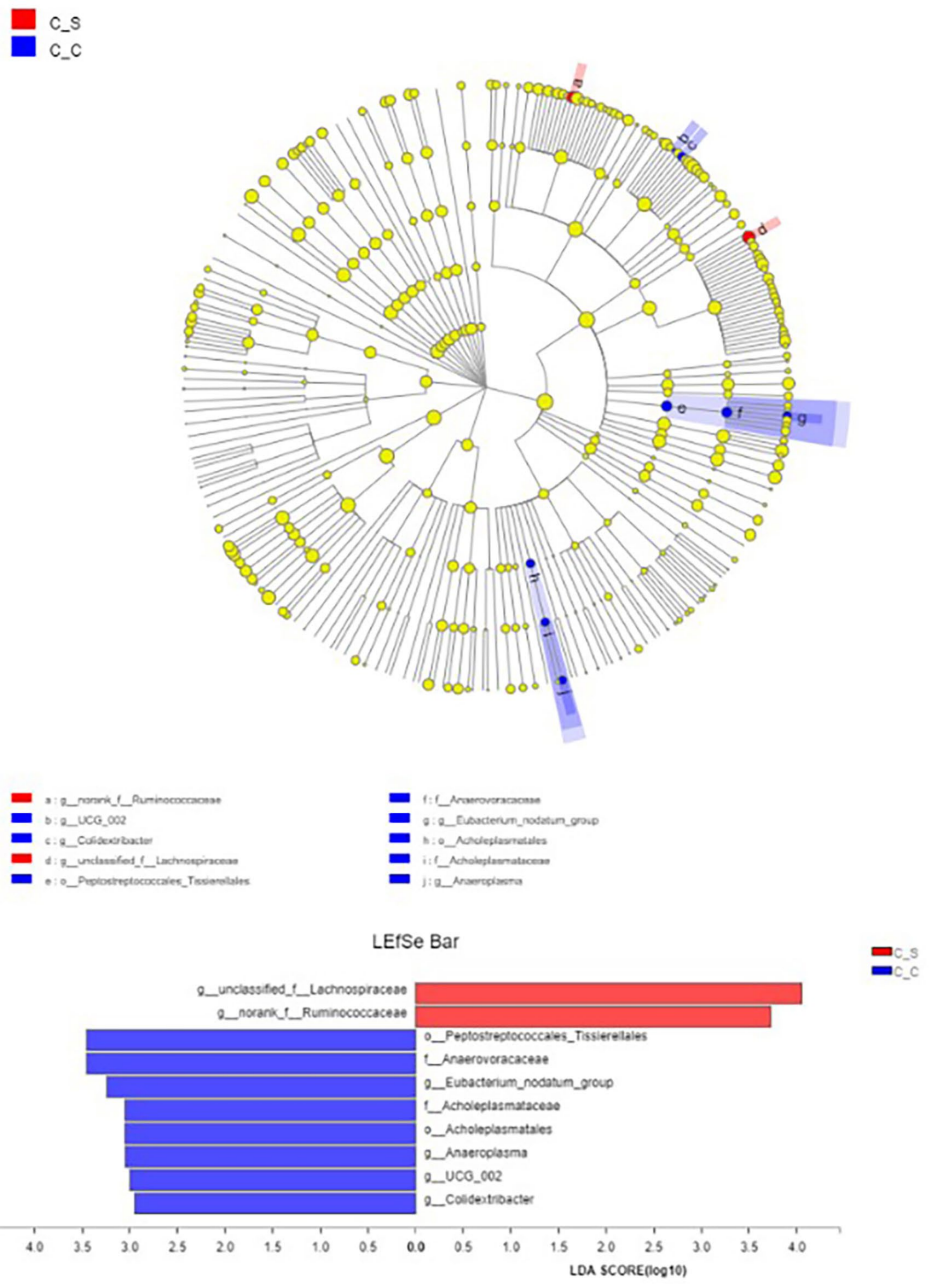
Column diagram of LDA value distribution (default set to 4) and evolutionary branch diagram. In the Figure, C▬S represents the HT group and C▬C represents the appropriate temperature group, *n*=6.

LEfSe (LDA Effect Size) analysis showed that the genera of Spirillaceae and Rumen Fungi played an important role in the heat stress group, while Colidextribacter, Eubacterium_nodatum_group and Anaeroplasma played an important role in the moderate temperature group, and the heat stress group played an important role in the heat stress group (Figure 10), while in the moderate temperature group, colonic bacilli, Escherichia coli and anaerobic mycoplasma played an important role in the heat stress group.

## Discussion

### Effects of heat stress on production performance, serum biochemical and hormonal parameters of meat rabbits

In recent years, the environmental problem has become an important factor affecting the development of the rabbit industry, especially the temperature problem. Studies have pointed out that meat rabbits exposed to high temperatures for a long time will inhibit the excitability of their feeding centers and decrease their food intake, resulting in insufficient supply of nutrients in the body. Heat stress reduces feed utilization and body weight gain by reducing feed intake and nutrient metabolism, which affects the production performance of meat rabbits. This is a typical manifestation of heat stress. In this study, it was found that under the condition of continuous high temperature, the average daily gain and average daily feed intake of meat rabbits decreased significantly. This is consistent with the results of previous studies (Liu et al., 2010; Anoh et al., 2020). A high-temperature environment has a great influence on the change of immune index, the increase in temperature will significantly inhibit the immune function (Thaxton et al., 1968), and its immune organ will change in response, and the index of the immune organ is an important index to reflect the development of the immune organ (Tang and Chen, 2016). In this study, it was found that the immune organ index of meat rabbits decreased significantly under high temperatures, which was consistent with the results of chicken research.

The changes in serum biochemical and hormonal parameters are important characteristics that reflect the changes in material metabolism and functional state of tissues and organs during heat stress, and it is found that serum biochemical indexes are related to productivity. HSP70 plays an important role in protecting antioxidant cells and reducing inflammation in animals (Farid et al., 2006; Zhou and Zhou, 2010). It is the main sign of biological heat shock response, and can still maintain a high measured value after the disappearance of the stressor, so the detection of HSP70 content can reflect the level of heat stress (Boyko, 2013). In this study, it was found that the content of HSP70 in the serum of meat rabbits increased under high temperatures, which was consistent with previous studies. The results showed that under the condition of high temperature, the content of alkaline phosphatase in meat rabbits increased, while the contents of total protein and serum glucose decreased, which was similar to the results of previous studies of Other species (Li et al., 2016). Under the condition of heat stress, meat rabbits produce available energy through glycogen decomposition to enhance their resistance to heat stress, so the supply of serum glucose can not be guaranteed, so the serum glucose concentration is reduced. When the meat rabbit is under heat stress, the weakening of thyroid activity leads to the obstruction of protein synthesis, which leads to the decrease of protein content in the blood. Heat stress caused cell damage, increased cell membrane permeability and increased enzyme content. It is the energy supply during stress, and the serum glucose decreases during chronic stress (Jia, 2012).

The results showed that when the ambient temperature was 34°C, the contents of T3 and T4 in the serum of meat rabbits decreased significantly, while the content of cortisol increased significantly (You et al., 1999). Thyroid hormone acts on almost all organs and tissues of the body, affecting growth, development, metabolism and other aspects. A large number of studies have shown that the increase in body temperature will reduce the secretory function of thyroid gland, reduce the level of thyroid hormone in blood during heat stress, inhibit the metabolic function of the body, and reduce thermogenesis (Cui and Gu, 2015). Heat stress modes significantly decreased the ADG, liver index and the contents of TP and GLU in serum, and increased the contents of cortisol and ALP in the serum of meat rabbits, which is similar to the research on broiler (Wang et al., 2003).

### Effects of heat stress on serum metabonomics in meat rabbits

In the state of heat stress, the body needs to use reserves to deal with heat stress, leading to metabolic disorders or the re-establishment of homeostasis. In this study, 39 kinds of differential metabolites were screened by multidimensional statistical analysis, which can effectively distinguish meat rabbits from the heat stress group and moderate temperature group. These potential biomarkers are involved in the phospholipid metabolism pathway, glyoxylic acid, and dicarboxylic acid metabolism, pyrimidine metabolism pathway, tricarboxylic acid cycle, nitrogen metabolism and other pathways, involving glucose metabolism, lipid metabolism, nucleic acid metabolism, amino acid metabolism, energy metabolism and translation process. The results of heat stress are consistent with those of broilers (Tang, 2015) and mice (Straadt et al., 2010; Ippolito et al., 2014). In this study, it was found that heat stress significantly decreased the levels of L-glutamine and L-malic acid in the blood of meat rabbits. Glutamine can produce glucose through gluconeogenesis (Anders, 1981; Mccauley et al., 1998; Shanware et al., 2011), and malic acid is an important intermediate in the tricarboxylic acid cycle (Jun-Lin et al., 2014; Wei et al., 2021). Glutamine is a necessary nutrient for intestinal mucosal cell metabolism, which plays a very important role in maintaining the integrity of the intestinal mucosal epithelial structure, improving the antioxidant capacity of the body and promoting protein synthesis (Zhangjun-Min, 2002).In addition, several recent reports have highlighted the functions of Gln metabolism in regulating oxidative stress resistance (Amoressánchez, 1999; Gao et al., 2009, p. 23).

Sphingosine and sphingosine-1-phosphate, which are involved in the pathway of phospholipid metabolism, were significantly down-regulated under heat stress. Sphingosine, also known as sphingosine, is one of the components of the cell membrane, while sphingosine-1-phosphate. Sphingosine-1-phosphate is a powerful signaling lipid molecule, which can regulate cell proliferation, regeneration, migration and intracellular calcium movement, expression of adhesion molecules and activation of monocytes adhering to endothelial cells (Spiegel and Milstien, 2003). Heat stress decreased the level of cytosine nucleosides involved in pyrimidine metabolism in the blood of meat rabbits, and L-glutamine was also involved in pyrimidine metabolism.

### Effects of heat stress on cecal microflora of meat rabbits

When the animals are in a healthy state, the species and number of intestinal microorganisms are relatively balanced, and there is a balance between the intestinal microflora host and the intestinal microenvironment. However, many external factors can break this balance, and it has been reported that diet, age and environmental factors can affect the intestinal flora structure of meat rabbits (Blas, 2013; Li et al., 2022). At present, it has been found that heat stress can cause changes in the structure of intestinal microflora in chickens and dairy cows (Song et al., 2014; Koch et al., 2019). The dominant bacteria in the cecum of meat rabbits were thick-walled bacteria and Bacteroides. The phylum of Proteus in the cecum of meat rabbit in the heat stress group was significantly higher than that in the temperature group, and it was gram-negative bacteria, including Escherichia coli, Salmonella, Helicobacter and other pathogenic bacteria. This shows that heat stress may increase the number of harmful bacteria in the intestinal tract of meat rabbits.

The dominant bacteria in the cecum were Ackermann (AKK) and Muri. Akk is a strictly anaerobic, non-mobile, non-spore-producing oval Gram-negative bacteria that can grow in the intestinal mucus layer and grow well using gastrointestinal mucin as the only carbon and nitrogen source. It can grow individually or in pairs, and can also grow in clusters in a medium containing mucin, thus settling in the intestines by competitive rejection and protecting the intestines from pathogens (Derrien et al., 2004). Although Akk bacteria use mucin as an energy source, a large number of observations have confirmed that Akk bacteria have a positive regulatory effect on intestinal mucus layer thickness and intestinal barrier integrity, which is closely related to energy metabolism, immune response and intestinal mucin secretion (Zhang et al., 2009; Png et al., 2010). Muribaculaceae, formerly known as S24-7, is a dominant family of Bacteroides in the intestinal tract, which has functional diversity in the degradation of complex carbohydrates. The Candidatus-saccharimonas microflora in the cecal flora of heat-stressed meat rabbits was significantly higher than that of the cecal samples in the moderate temperature group. Studies have found that Candidatus-saccharimonas may be associated with inflammatory diseases (Cruz et al., 2020). In this study, it was found that heat stress had a certain effect on the structure of cecal flora in meat rabbits, which may cause intestinal inflammation and increase the abundance of harmful bacteria.

## Conclusion

In summary,combined physiological, UHPLC–MS/MS-based metabolomics, and 16S rRNA microbiome analyses were performed to investigate metabolic and gut microbiota responses in the meat rabbits to continuous high-temperature exposure for 14 days. The results revealed that heat stress significantly reduced the production performance of meat rabbits and destroyed the blood biochemical balance of meat rabbits. There were 9 kinds of differential metabolites could be used as biomarkers of heat stress in meat rabbits. Heat stress may affect glucose and fat metabolism in meat rabbits by modulating the gut microflora. These investigations provide new insights into the metabolism mechanisms of meat rabbits to heat stress and contribute to a better understanding of the adverse effects of heat stress on rabbits.

## Data availability statement

The datasets presented in this study can be found in the Supplementary material and online repositories. The names of the repository/repositories and accession number(s) of 16s sequencing data contained in our article can be found at: https://www.ncbi.nlm.nih.gov/, BioProject PRJNA899341 with corresponding BioSample Accessions SAMN31652990-SAMN31653001.

## Ethics statement

All study procedures were approved by the Shandong Agricultural University Animal Care and Use Committee (SDAUA-2017-057) and were following the Guidelines for Experimental Animals established by the Ministry of Science and Technology (Beijing, China).

## Author contributions

HL and FL planned the experiment and finalized the draft of the manuscript with consent from all contributing authors. HL, BZ, and FL carried out the experiment, finalized, and analyzed the collected data. TY, HZ, and LL assisted with manuscript editing and language calibration. All authors contributed to the article and approved the submitted version.

## Funding

This work was supported by China Agriculture Research System of MOF and MARA (CARS-43-B-1), Taishan Industry Leadership Project (TSCY20190107), National Natural Science Foundation of China (31972594), and Funds of Shandong “Double Tops” Program.

## Conflict of interest

The authors declare that the research was conducted in the absence of any commercial or financial relationships that could be construed as a potential conflict of interest.

## Publisher’s note

All claims expressed in this article are solely those of the authors and do not necessarily represent those of their affiliated organizations, or those of the publisher, the editors and the reviewers. Any product that may be evaluated in this article, or claim that may be made by its manufacturer, is not guaranteed or endorsed by the publisher.

## Footnotes

^1^ http://www.genome.jp/kegg/

^2^ http://www.hmdb.ca/

^3^ http://www.lipidmaps.org/
