# Supplementary material for: Effects of heat stress on growth performance, carcass traits, serum metabolism, and intestinal microflora of meat rabbits
Source: Front Microbiol. 2022 Nov 28;13:998095. doi: 10.3389/fmicb.2022.998095 (PMC9743647; doi:10.3389/fmicb.2022.998095)
Supplement: Supplementary file 1 [file Data_Sheet_1.docx]

Supplementary Material S1

Metabonomic analysis based on UPLC-MS/MS

The serum samples were taken from the refrigerator at-80 ℃ and thawed in the refrigerator at 4 ℃. Six samples were selected for each group. 100 μ L and 400 μL pre-cooled 80% methanol containing 0.1% formic acid were mixed by rotation method, and the samples were incubated with 5min on ice. Under the condition of 15000g 10min at 4 °C, the supernatant was injected into the sample by centrifugation and analyzed by LC-MS/MS system.

LC-MS/MS analyses were performed using a Vanquish UHPLC system (Thermo Fisher) coupled with an Orbitrap Q Exactive series mass spectrometer (Thermo Fisher). Samples were injected onto an Hyperil Gold column (100×2.1 mm, 1.9μm) using a 16- min linear gradient at a flow rate of 0.2mL/min. The eluents for the positive polarity mode were eluent A (0.1% FA in Water) and eluent B (Methanol). The eluents for the negative polarity mode were eluent A (5 mM ammonium acetate, pH 9.0) and eluent B (Methanol).The solvent gradient was set as follows: 2% B, 1.5 min; 2-100% B, 12.0 min; 100% B, 14.0 min;100-2% B, 14.1 min;2% B, 17 min. Q Exactive series mass spectrometer was operated in positive/negative polarity mode with a spray voltage of 3.2 kV, capillary temperature of 320°C, sheath gas flow rate of 35 arb, and aux gas flow rate of 10 arb.

The raw data files generated by UHPLC-MS/MS were processed using the Compound Discoverer 3.1 (CD3.1, Thermo Fisher) to perform peak alignment, peak picking, and quantitation for each metabolite. The main parameters were set as follows: retention time tolerance, 0.2 minutes; actual mass tolerance, 5ppm; signal intensity tolerance, 30%; signal/noise ratio, 3; and minimum intensity, 100000. After that, peak intensities were normalized to the total spectral intensity. The normalized data were used to predict the molecular formula based on additive ions, molecular ion peaks, and fragment ions. And then peaks were matched with the mzCloud (<https://www.mzcloud.org/>), mzVault, and MassList database to obtained accurate qualitative and relative quantitative results. Statistical analyses were performed using the statistical software R (R version R-3.4.3), Python (Python 2.7.6 version), and CentOS (CentOS release 6.6), When data were not normally distributed, normal transformations were attempted using of area normalization method. 4. Data Analysis These metabolites were annotated using the Kyoto Encyclopedia of Genes and Genomes (KEGG) database (<http://www.genome.jp/kegg/>), Human Metabolome database (HMDB) (<http://www.hmdb.ca/>), and Lipidmaps database (http://www.lipidmaps.org/). Principal components analysis (PCA), Partial least squares discriminant analysis (PLS‐DA), and Orthogonal partial least-squares discrimination analysis (OPLS-DA) was performed at metaX (a flexible and comprehensive software for processing metabolomics data). We applied univariate analysis (t-test) to calculate the statistical significance (P-value). The metabolites with VIP > 1 and *P*-value< 0.05 and Fold change (FC)≥1.2 or FC≤ 0.83 were considered to be differential metabolites. Volcano plots were used to filter metabolites of interest which based on Log2(FC) and -log10(P-value) of metabolites. For clustering heat maps, the data were normalized using z-scores of the intensity areas of differential metabolites and were ploted by Pheatmap package in R language. The correlation between differential metabolites were analyzed by cor in R language (method = pearson). Statistically significant of correlation between differential metabolites were calculated by cor.mtest in R language. *P*-value < 0.05 was considered as statistically significant and correlation plots were ploted by corrplot package in R language. The functions of these metabolites and metabolic pathways were studied using the KEGG database. The metabolic pathway enrichment of differential metabolites were performed, when ratio were satisfied by x/n > y/N, metabolic pathway were considered as enrichment, when P-value of metabolic pathway<0.05, metabolic pathway were considered as statistically significant enrichment

**Gut microbiomics**

Microbial community genomic DNA was extracted from cecal contents samples using the E.Z.N.A.® soil DNA Kit (Omega Bio-tek, Norcross, GA, U.S.) according to manufacturer’s instructions.The DNA extract was checked on 1% agarose gel, and DNA concentration and purity were determined with NanoDrop 2000 UV-vis spectrophotometer (Thermo Scientific, Wilmington, USA). The hypervariable region V3-V4 of the bacterial 16S rRNA gene were amplified with primer pairs 338F (5'-ACTCCTACGGGAGGCAGCAG-3') and 806R(5'-GGACTACHVGGGTWTCTAAT-3') by an ABI GeneAmp® 9700 PCR thermocycler (ABI, CA, USA).

The PCR amplification of 16S rRNA gene was performed as follows: initial denaturation at 95 ℃ for 3 min, followed by 27 cycles of denaturing at 95 ℃ for 30 s, annealing at 55 ℃ for 30 s and extension at 72 ℃for 45 s, and single extension at 72 ℃ for 10 min, and end at 4 ℃. The PCR mixtures contain 5 × TransStart FastPfu buffer 4 μL, 2.5 mM dNTPs 2 μL , forward primer (5 μM) 0.8 μL, reverse primer (5 μM) 0.8 μL, TransStart FastPfu DNA Polymerase 0.4 μL, template DNA 10 ng, and finally ddH2O up to 20 μL. PCR reactions were performed in triplicate. The PCR product was extracted from 2% agarose gel and purified using the AxyPrep DNA Gel Extraction Kit (Axygen Biosciences, Union City, CA, USA) according to manufacturer’s instructions and quantified using Quantus™ Fluorometer (Promega, USA).

Purified amplicons were pooled in equimolar and paired-end sequenced on an Illumina NovaSeq PE250 platform (Illumina, San Diego,USA) according to the standard protocols by Majorbio Bio-Pharm Technology Co. Ltd. (Shanghai, China). The raw reads were deposited into the NCBI Sequence Read Archive (SRA) database.

The raw 16S rRNA gene sequencing reads were demultiplexed, quality-filtered by fastp version 0.20.0 and merged by FLASH version 1.2.7 with the following criteria: (i) the 300 bp reads were truncated at any site receiving an average quality score of <20 over a 50 bp sliding window, and the truncated reads shorter than 50 bp were discarded, reads containing ambiguous characters were also discarded; (ii) only overlapping sequences longer than 10 bp were assembled according to their overlapped sequence. The maximum mismatch ratio of overlap region is 0.2. Reads that could not be assembled were discarded; (iii) Samples were distinguished according to the barcode and primers, and the sequence direction was adjusted, exact barcode matching, 2 nucleotide mismatch in primer matching.

Operational taxonomic units (OTUs) with 97% similarity cutoff were clustered using UPARSE version 7.1, and chimeric sequences were identified and removed. The taxonomy of each OTU representative sequence was analyzed by RDP Classifier version 2.2 against the 16S rRNA database (eg. Silva v138) using confidence threshold of 0.7.
